# TNF-α-induced miR-155 regulates IL-6 signaling in rheumatoid synovial fibroblasts

**DOI:** 10.1186/s13104-017-2715-5

**Published:** 2017-08-14

**Authors:** Kiyoshi Migita, Nozomi Iwanaga, Yasumori Izumi, Chieko Kawahara, Kenji Kumagai, Tadashi Nakamura, Tomohiro Koga, Atsushi Kawakami

**Affiliations:** 1 Department of Rheumatology, Fukushima Medical University, Hikarigaoka 1, Fukushima, 960-1295 Japan; 2 Department of Rheumatology and Clinical Research Center, NHO Nagasaki Medical Center, Omura, Nagasaki Japan; 3 Department of Orthopedics, Clinical Research Center, NHO Nagasaki Medical Center, Omura, Nagasaki Japan; 4 Department of Rheumatology, Shinto Kumamoto Hospital, Kumamoto, Kumamoto Japan; 5 Department of Rheumatology, Nagasaki University Graduate School of Biomedical Sciences, Nagasaki, Nagasaki Japan

**Keywords:** Cytokines, Interleukin-6, MicroRNAs, Rheumatoid arthritis, Synovial fibroblasts, Tumor necrosis factor-α

## Abstract

**Background:**

MicroRNAs (miRNAs) are important regulators of a variety of inflammatory mediators. The present study was undertaken to elucidate the role of miRNAs in the rheumatoid cytokine network.

**Methods:**

We analyzed miRNA expression in rheumatoid synovial fibroblasts (RASFs). miRNA array-based screening was used to identify miRNAs differentially expressed between tumor necrosis factor-α (TNF-α)-activated RASFs and untreated RASFs. Transfection of RASFs with miR-155 was used to analyze the function of miR-155. Real-time polymerase chain reaction (PCR) was used to measure the levels of miR-155 in RASFs.

**Results:**

miRNA microarray analysis revealed that miR-155-5p was the most highly induced miRNA in TNF-α-stimulated RASFs. TNF-α-induced miR-155 expression in RASFs was time-dependent and TNFα dose-dependent, whereas, IL-6 stimulation did not affect miR-155 expression in RASFs. Transfection of miR-155 mimics into RASFs resulted in the decrease JAK2/STAT3 phosphorylation in IL-6-treated RASFs.

**Conclusions:**

The current results demonstrate that TNF-α modulated miRNA expressions in RASFs. Our data showed that miR-155, which is highly induced by TNF-α stimulation, inhibits IL-6-mediated JAK2/STAT3 activation in RASFs. These findings suggest that miR-155 contributes to the cross-regulation between TNF-α and IL-6-mediated inflammatory pathways in RA.

## Background

Potential triggers of rheumatoid arthritis (RA) include cytokines and dysregulation of the cytokine network can result in uncontrolled inflammation leading to rheumatoid arthritis [1].

TNF-α and IL-6 are key inflammatory cytokines implicated in the pathogenesis of RA [2]. Targeted blockade of TNF-α and IL-6 is effective for refractory RA patients [3]. TNF-α secretion from synovial macrophages may be a major player in rheumatoid synovitis [4]. However, TNF-α-triggered cytokine signaling in rheumatoid inflammation is not completely understood, and the elucidation of endogenous positive and negative modulators of cytokine signaling is needed for a more effective therapeutic approach [5].

miRNAs play important roles in inflammatory processes and miRNA-based gene therapies targeting deregulated miRNAs have great potential [6]. Important features of miRNAs include their redundancy to target sequences in the 3′-untranslated regions of messenger RNAs [7]. These unique properties prompted us to speculate that miRNAs can regulate the cytokine-network during the pathogenesis of RA [8]. Recent studies have identified altered miRNA expression in RA patients [9]. These investigations demonstrated increased expression of miR-155 and miR-146a in both rheumatoid synovial fibroblasts and synovial tissues compared to osteoarthritis (OA) patients [10]. These findings suggest that altered miRNA expression contributes to the rheumatoid inflammatory process by affecting resident synovial cells of the rheumatoid joint and their cytokine network [11]. We hypothesized that TNF-α modulates the inflammatory signaling pathway by regulating other cytokines via miRNAs.

## Methods

### Reagents

Phospho-specific and pan antibodies against JAK-1 (Tyr1022/1023), JAK-2 (Tyr1007/1008), STAT-1 (Tyr701) and STAT-3 (Tyr705) were purchased from Cell Signaling Technology (Beverly, MA).

### Preparation of RASFs

Synovial tissue was obtained from patients with RA at the time of total joint replacement. Synovium was minced and incubated with 1 mg/ml collagenase type VIII (Sigma-Aldrich, St. Louis, MO) in serum-free RPMI 1640 medium (Life Technologies, Grand Island, NY) for 1 h at 37 °C, filtered, extensively washed, and cultured in DMEM (Life Technologies) supplemented with 10% FBS (Gibco, 10270106) in 12 well flat bottom cell culture plates (Falcon^®^ #353043). Synovial fibroblasts (SFs) were used from passages 4 through 7th. The expression of CD45 on synovial fibroblasts was measured by flow cytometry using a Coulter Epics XL flow cytometer (Beckman Coulter, Brea, CA, USA) using Expo32 ADC analysis software (Beckman Coulter) and we confirmed that synovial fibroblasts were negative for CD45 (<1% CD45-positive). Synovial tissue samples were obtained from three female patients (mean age: 62.3 years old) with RA during synovectomy.

### Microarray analysis for miRNAs

Small RNA was isolated from RASFs using QIAzol reagent according to the manufacturer’s instructions (Qiagen, Hilden, Germany). Microarray analysis was performed to evaluate miRNA expression patterns in RASFs using 3D-Gene miRNA Oligo chips according to the manufacturer’s instructions (Toray Industries, Inc. Tokyo, Japan). Small RNAs from RASFs were labelled using the miRCURY LNA™ microRNA Array Power Labelling Kit (Exiqon. Palm Beach FL, USA) and analyzed using 3D-Gene miRNA Oligo chips Ver. 17.0 (Toray Industries Inc.) containing more than 1700 antisense probes printed in duplicate spots, according to the manufacturer’s instructions. Signals were analyzed using the 3D-Gene Scanner 3000 (Toray Industries Inc. Tokyo, Japan) and analyzed using 3D-Gene Extraction software (Toray Industries Inc., Tokyo, Japan). A relative expression level of a given miRNA was calculated by comparing the signal intensities of the valid spots throughout the microarray experiments. The Normalized data were globally normalized per array, such that the median of the signal intensity was adjusted to 25.

### QuantitativePCR (qPCR) analysis for miR-155-5p

Small RNA was isolated from RASFs using Qiazol reagent according to the manufacturer’s instructions (Qiagen, Limburg, The Netherlands). The RNA quality was assessed by microcapillary electrophoresis (2100 BioAnalyser, Agilent Technologies, Waldbronn, Germany). cDNA was reverse transcribed from 2.5 µl RNA using the TaqMan miRNA reverse transcription kit (TaqManR MicroRNA Reverse Transcription Kit, AppliedBiosystems^®^). qRT-PCR for the detection of hsa-miR-155-5p was carried out in 20 µl PCR reactions using the TaqMan MicroRNA assay with the StepOnePlus detection system (Applied Biosystems) at 50 °C for 2 min then 95 °C for 10 min, followed by 40 cycles of 95 °C for 15 s and 60 °C for 1 min. Expression of RNU6B were used as internal controls to normalize each mRNA and miRNA expression, respectively. The comparative threshold cycle (Ct) method was used for relative quantification of the miRNA. Differences in the Ct values (ΔCt) between the tested miR-155 and RNU6B cDNA were calculated to determine the relative expression levels, using the following formula: ΔΔCt = ΔCt of the tested sample—ΔCt of the control sample. The value of each control sample was set at 1 and was used to calculate the fold change in target genes.

### Transfection of miR-155

RASFs (n = 3) were transfected in 12-well plates (5 × 10^4^ cells/well) using Lipofectamine 2000 reagent (Invitrogen) according to the manufacturer’s protocol, with 100 nM (final concentration) of synthetic miR-155 precursory molecule (miR-155 mimics) or a scrambled control (negative control, Thermo Fisher Scientist Inc. Waltham, MA, USA).

### Cell lysis and Western blotting

Micro-RNAs-transfected-RA-FLS were serum-starved (12 h), then stimulated with IL-6 (50 ng/ml) and sIL-6R (50 ng/ml) for indicated time and the cells were washed by ice-cold PBS and lysed with RIPA Buffer (Sigma-Aldrich, R0278) supplemented with 1.0 mM sodium orthovanadate, 10 μg/ml aprotinin and 10 μg/ml leupeptin) for 20 min at 4 °C. Insoluble material was removed by centrifugation at 15,000×*g* for 15 min at 4 °C. The supernatant was saved and the protein concentration was determined using the Bio-Rad protein assay kit (Bio Rad, Hercules, CA, 5000001). An identical amount of protein (30 μg) for each lysate was subjected to 10% SDS–polyacrylamide gel electrophoresis, and then transferred to a nitrocellulose membrane. Western blot analysis using phospho-specific anti-JAKs, and STATs antibodies was performed with an ECL advance western blotting detection kit (GE Health RPN2135).

### Statistical analysis

Differences between groups were examined for statistical significance using Student’s two-tailed t-test. *P* values less than 0.05 were considered statistically significance.

## Findings

### miRNA expression profile in TNF-α-activated RASFs

**Fig. 1:**
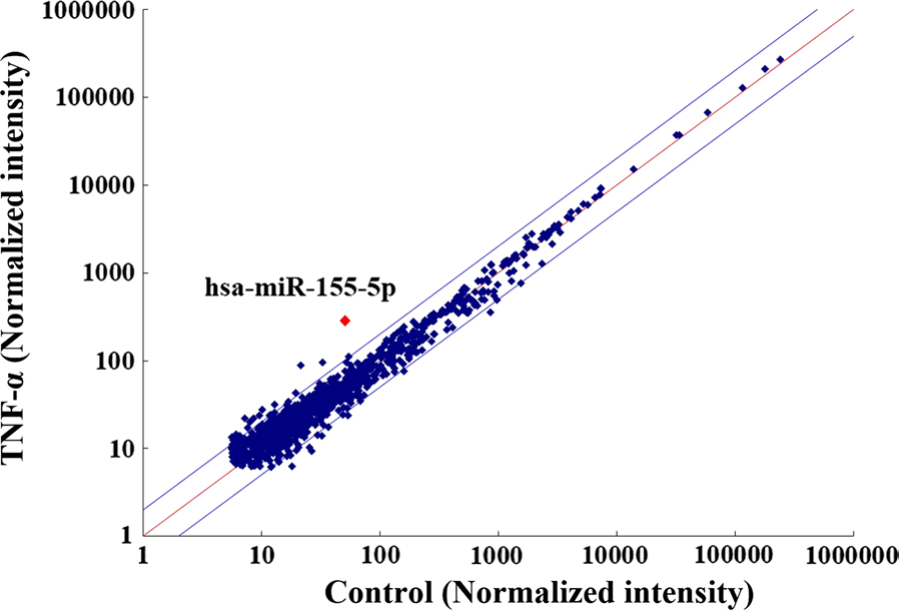
Comparison of miRNA expressions in TNF-α stimulated or unstimulated RASFs by miRNA microarray. Comparison of normalized signal intensities of various miRNAs in small RNAs isolated from RASFs. *X axis* represents unstimulated RASFs and *Y axis* represent TNF-α stimulated RASFs

**Fig. 2:**
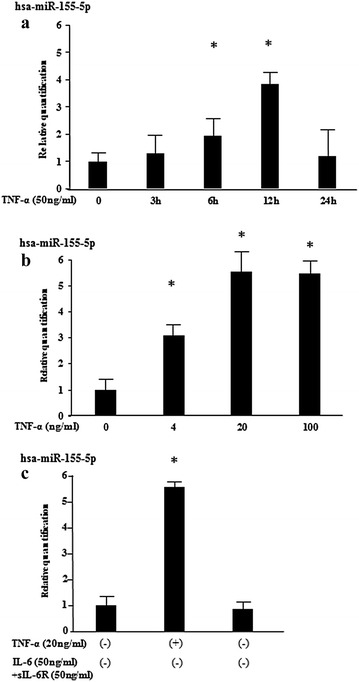
TNF-α induces miR-155 expression in RASFs. **a** RASFs were stimulated with TNF-α (50 ng/ml) for the indicated periods and relative expression of miR-155 were analyzed by qRT-PCR (n = 3). Values represent the mean ± SD of three independent experiments. *p < 0.005 as compared with the value in unstimulated cells. Three experiments were performed using RASFs isolated from three different RA patients and a representative result is shown. **b** RASFs were stimulated with various concentrations of TNF-α for 12 h and relative expression of miR-155 were analyzed by qRT-PCR (n = 3). Values represent the mean ± SD of three independent experiments. *p < 0.005 as compared with the value in unstimulated cells. Three experiments were performed using RASFs isolated from three different RA patients and a representative result is shown. **c** RASFs were stimulated with IL-6 (50 ng/ml) plus sIL-6R (50 ng/ml) or TNF-α (50 ng/ml) for 12 h and relative expression of miR-155 were analyzed by qRT-PCR (n = 3). Values represent the mean ± SD of three independent experiments. *p < 0.001 as compared with the value in unstimulated cells. Three experiments were performed using RASFs isolated from three different RA patients and a representative result is shown

As an initial experiment, to determine which miRNAs are expressed in TNF-α-exposed rheumatoid synoviocytes (RASFs), we isolated miRNAs from TNFα-treated or untreated RASFs and screened them against a miRNA array consisting of 3450 miRNAs. A comparison between TNF-α stimulated and unstimulated RASFs identified 10 miRNAs that were up-regulated by more than twofold (Table 1). Among these isolated miRNAs, miR-155-5p was most highly up-reglated in TNF-α-stimulated RASFs (Table 1; Fig. 1). To confirm the array results, we examined miRNA-155 expression in TNF-α-stimulated RASFs isolated from three RA patients using quantitative real-time PCR using. We treated the RASFs in vitro with TNF-α (50 ng/ml) for 3–24 h, and then assessed miRNA-155 expression by real-time PCR. We observed significant up-regulation of miR-155 6 h after TNF-α stimulation that reached a plateau after 12 h (Fig. 2a). Additionally, TNFα-stimulated RASFs secreted miR-155-5p into the culture medium in a dose-dependent manner (Fig. 2b). We then investigated the regulation of miR-155 expression by another cytokine, IL-6. In IL-6 trans-signaling system, IL-6/soluble IL-6 receptor (sIL-6R) complex resulted in activation of JAK/STAT pathway in rheumatoid synovial fibroblasts [12]. It was also demonstrated that without sIL-6R, IL-6 stimulation alone could not induce various genes expressions in rheumatoid synovial fibroblast [13]. Therefore, we stimulated synovial fibroblast with IL-6 in the presence of sIL-6R at the optional concentrations as described previously [13]. As shown in Fig. 2c, miR-155 expression in RASFs was not induced by IL-6 stimulation in the presence of sIL-6R.

**Table 1:** A differentially expression miRNAs between control and TNFα-stimulated RASFs

Up			Down		
No.	miR_name	Fold change	No.	miR_name	Fold change
1	hsa-miR-155-5p	5.63	1	hsa-miR-637	0.35
2	hsa-miR-2467-3p	4.17	2	hsa-miR-4675	0.35
3	hsa-miR-933	3.07	3	hsa-miR-4746-3p	0.40
4	hsa-miR-4521	2.92	4	hsa-miR-6131	0.41
5	hsa-miR-4708-3p	2.73	5	hsa-miR-222-5p	0.43
6	hsa-miR-26b-3p	2.57	6	hsa-miR-1298-5p	0.44
7	hsa-miR-3193	2.51	7	hsa-miR-6875-5p	0.47
8	hsa-miR-4313	2.46	8	hsa-miR-6792-5p	0.47
9	hsa-miR-6715b-5p	2.44	9	hsa-miR-6858-5p	0.48
10	hsa-miR-33a-5p	2.42	10	hsa-miR-1470	0.48

miRNAs which were upregulated (>2-fold) or downregulated (<0.5-fold) from basal levels by TNF-α stimulation were listed

### miR-155-5p regulates IL-6-mediated signaling in RASFs

**Fig. 3:**
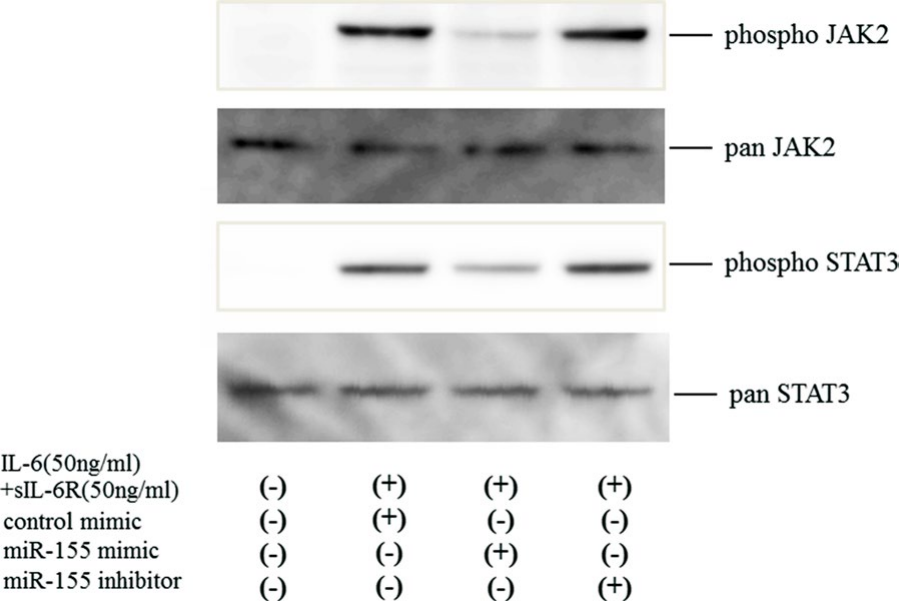
Effects of miR-155 on JAKs/STATs phosphorylation in IL-6-stimulated RASFs (short time course). RASFs in a density of 5 × 10^4^ cells/well in 12-well culture plates were transfected with miR-155 mimics or control for 48 h, serum-starved (12 h) and stimulated with IL-6 (50 ng/ml) plus sIL-6R (50 ng/ml) for the indicated periods. Phosphorylation of JAK1, JAK2, STAT1, and STAT3 were determined by Western blotting using phospho-specific or pan antibodies against JAK2, STAT1 and STAT3. Phosphorylation of JAK1 was barely detected (data not shown). Three experiments were performed using RASFs isolated from three different RA patients and a representative result is shown

To investigate the roles of miR-155-5p in IL-6-mediated signal transduction pathway, we transfected RASFs with miR-155 mimic before stimulation with IL-6 plus soluble IL-6 receptor (sIL-6R). RASFs pretreated with miR-155 were stimulated by IL-6 [IL-6 plus soluble IL-6 receptor (sIL-6R)] for 20 min. The phosphorylation of JAK1/2 and STAT1/STAT3 was investigated by immunoblot analysis. IL-6 stimulation induced JAK2 and STAT3 phosphorylation in RASFs, whereas, JAK1 and STAT1 phosphorylation was barely detected. The levels of phosphorylation of JAK2 and STAT3, which were rapidly induced by IL-6 stimulation, were down-regulated in RASFs transfected with miR-155 mimics. Whereas, the phosphorylation levels of JAK2/STAT3 were not affected in RASFs transfected with control mimics or miR-155 inhibitors (Fig. 3). These data indicate that TNF-α-induced over-expression of miR-155 resulted in the down-regulation of IL-6-induced JAK2/STAT3 activation in RASFs.

## Discussion

TNF-α is an important pathogenic cytokine in RA and epigenetic modulation by TNF-α could be implicated in rheumatoid synovitis [14]. The miRNA network is emerging as an important factor in the pathophysiology of autoimmune and inflammatory disorders [11]. Previous studies have reported aberrant expressions of miRNAs in blood or joint tissues and suggested their pathogenic roles in the inflammatory processes in RA [8]. The principal purpose of this study was to determine the mechanism and biological consequences of TNF-α against IL-6 signaling in RASFs. The results demonstrated an interaction relationship between TNF-α and subsequent IL-6 signaling, which may be attributable to TNF-α-induced miR-155-5p. This TNF-α-mediated epigenetic modulation may contribute to the cross-regulation between TNF-α and IL-6.

IL-6 is a pivotal proinflammatory factor in rheumatoid synovitis [15]. However, the mechanism by which IL-6 signaling is regulated and the role of TNF-α in this cytokine pathway in the rheumatoid synovium is not completely understood. Here, we have identified miR-155 as an important player in IL-6 mediated signal transduction pathway of rheumatoid synovial fibroblasts. miR-155, a typical multi-functional miRNA, is emerging as a novel regulator involved in inflammatory signal pathway [16]. To investigate the roles of miR-155 in IL-6-meliated signal transduction pathway, we transfected rheumatoid synovial fibroblasts with miR-155 mimic before stimulation with IL-6 plus soluble IL-6 receptor (sIL-6R). Our results demonstrated that miR-155 over-expression regulates IL-6 signaling by preventing JAK2/STA3 activation in rheumatoid synovial fibroblasts. This constitutes a negative-feedback loop that regulates the persistent IL6-mediated inflammatory cascades in rheumatoid synovitis.

miR-155, a typical multi-functional miRNA, is emerging as a novel regulator involved in inflammatory signal pathway [16]. Diverse cellular activities of miR-155 have been described, including involvement in oncogenesis [17]. McInnes et al. demonstrated that miR-155 is up-regulated in rheumatoid synovial tissues and that this up-regulation targeted an inhibitor of inflammation, SHIP-I, which leads to increased levels of proinflammatory cytokines [18]. The fact that miR-155 expression is induced by TNF-α suggests that miR-155 modulates TNF-α-triggered inflammatory cascades in rheumatoid synovitis. We have demonstrated that miR-155 could be implicated with IL-6 signaling pathway by suppressing JAK2/STAT3 activation in RASFs. During the interaction between the TNF-α and IL-6, up-regulated miR-155 expression in TNF-α-induced rheumatoid synovium may negatively regulate IL-6-triggered proinflammatory pathway by preventing JAK2/STAT3 activation. Up-regulated miR-155 controls the expression of MMP-3 and MMP-1 in rheumatoid synovial cells suggesting that miR-155 may be involved in the modulation of rheumatoid inflammatory processes [10]. Our data also suggest that miR-155 might provide a negative feedback loop in the IL-6-mediated inflammatory cascade in rheumatoid inflammatory processes.

Intracellular signaling pathways, that concurrently activated by the inflammatory stimulus, often interact with one another cross-regulatory feedback mechanism [19]. Normally, JAK/STAT pathway induces the expression of SOCS1, which turns off IL-6 signaling by acting through the tyrosine motif 759 of gp130 and blocks JAK and subsequent STAT3 activation [20]. Epigenetic silencing of SOCS1 via miR-155 over-expression was shown to permit constitutive IL-6/STAT3 signaling in lymphoid cells [21]. In contrast, miR-155 was demonstrated to be a part of negative feed-back loop in inflammatory cytokine networks. For example, miR-155 expression negatively regulates the inflammatory cytokines cascades by inhibiting TAK1-binding protein 2 (TAB 2) [22]. The mechanism by which IL-6 signaling is regulated by miR-155 in the RASFs is not elucidated in this study and further investigations should be needed.

## Conclusion

Our data demonstrates that TNF-α is a potent inducer of miR-155 in RASFs. Furthermore, miR-155 over-expression in rheumatoid synoviocytes resulted in the inhibition of IL-6-mediated JAKs/STAT3 activation. These findings suggest that the epigenetic cross-regulatory mechanism exists in the interaction of TNF-α/IL-6 of rheumatoid synovial inflammation.

## Abbreviations

IL-6: interleukin-6
JAK: Janus kinas
miRNAs: microRNAs
RASFs: rheumatoid synovial fibroblasts
STAT: signal transducer and activator of transcription
TNF-α: tumor necrosis factor-α
